# Neoadjuvant Sintilimab Plus Chemotherapy in Resectable Locally Advanced Esophageal Squamous Cell Carcinoma

**DOI:** 10.3389/fonc.2022.864533

**Published:** 2022-04-29

**Authors:** Huilai Lv, Yang Tian, Jiachen Li, Chao Huang, Bokang Sun, Chunyue Gai, Zhenhua Li, Ziqiang Tian

**Affiliations:** ^1^ Department of Thoracic Surgery, The Fourth Hospital of Hebei Medical University, Shijiazhuang, China; ^2^ Department of Thoracic and Cardiac Surgery, Hangzhou Hospital of Traditional Chinese Medicine, Hangzhou, China

**Keywords:** esophageal squamous cell carcinoma, pathologic complete response, combination therapy, immune checkpoint inhibitors, sintilimab

## Abstract

**Background:**

Neoadjuvant chemotherapy (nCT) and chemoradiotherapy (nCRT) are the standard treatments in patients with resectable locally advanced esophageal squamous cell carcinoma (ESCC). Adding PD-1 inhibitor to the chemotherapy has shown significant clinical benefits in first-line treatment of advanced ESCC. This study evaluated the efficacy and safety of neoadjuvant sintilimab plus chemotherapy in patients with resectable locally advanced ESCC.

**Methods:**

The clinical data of 96 patients with resectable locally advanced ESCC, treated with sintilimab plus chemotherapy followed by esophagectomy, were reviewed. The pathologic complete response (pCR) rate, major pathological response (MPR) rate, R0 resection rate, tumor downstaging, survival, and safety were retrospectively analyzed.

**Results:**

Patients were between the ages of 43 and 78 years (interquartile range [IQR], 60–69 years). Forty (41.7%) were diagnosed with stage II ESCC, 52 (54.2%) with stage III, and 4 (4.2%) with stage IVA. Sixty-seven (69.8%) were male, and 84 (87.5%) patients had an ECOG PS of ≤1. Forty-eight (50.0%) patients received 3–4 cycles of the neoadjuvant treatment. Twenty-nine (30.2%) patients obtained pCR, and MPR was achieved in 60 (62.5%) patients. The R0 resection rate was 99%. Eighty (83.3%) patients achieved clinical downstaging, and 71 (74.0%) achieved pathological downstaging. The median follow-up was 8.9 months, and 1-year DFS rate was 95.2% (95% CI, 88.8%–100%). Grade 3–4 TRAEs occurred in 12 (12.5%) patients, and the incidence of grade 3–4 surgical complications was 2.1%. No deaths were reported.

**Conclusion:**

These real-world data revealed that neoadjuvant sintilimab plus chemotherapy could provide encouraging pCR with good tolerability for resectable locally advanced ESCC, and this regimen warrants further exploration in prospective clinical studies.

## Introduction

Esophageal cancer (EC) is the seventh most common cancer and the sixth most common cause of cancer mortality worldwide (1). More than half of new and fatal cases of esophageal cancer in the world occur in China (1). In China, EC is the sixth leading type of cancer and the fourth most common cause of death from cancer, with approximately 320,000 new cases and 300,000 deaths in 2020 (2). Histologically, esophageal squamous cell carcinoma (ESCC) is more prevalent in China, accounting for 90% of all ECs (3, 4). For patients with locally advanced esophageal cancer, surgical resection remains the mainstay of treatment. However, surgical resection with no additional therapies is accompanied by high recurrence or metastasis rates with poor survival (5–7). In light of the improved postoperative survival in patients with locally advanced ESCC, neoadjuvant treatment combined with surgery was recommended by the National Comprehensive Cancer Network (NCCN) guidelines (8) and the Chinese Society of Clinical Oncology (CSCO) guidelines (9). As demonstrated by previous clinical studies, neoadjuvant chemotherapy and neoadjuvant chemoradiotherapy provide significant survival benefits and have therefore been routinely adopted (10–16).

Preclinical studies have demonstrated that chemotherapeutic agents can exert immunostimulatory effects, either by activating effector cells and/or inhibiting immunosuppressive cells in the tumor microenvironment (TME) or by increasing immunogenicity and T-cell infiltration (17–19). Clinically, Programmed Cell Death Ligand 1 (PD-L1) inhibitors combined with chemotherapy have shown promising clinical benefits and represent a new first-line (1L) treatment option for patients with advanced ESCC. Based on the favorable data from the KEYNOTE-590 Clinical Trial (20), pembrolizumab combined with chemotherapy were recently approved by the US Food and Drug Administration (FDA), the National Medical Products Administration of China (NMPA), and other regulatory agencies in the first-line setting for advanced esophageal and gastroesophageal junction (GEJ) carcinoma. Sintilimab is a selective anti-PD-1 antibody that inhibits the interaction between PD-1 and its ligands, and sintilimab combined with chemotherapy showed a significant OS benefit in patients with advanced or metastatic ESSC in the ORIENT-15 study (21).

Although the evidence of anti-PD-1 treatment in combination with chemotherapy is strong in advanced ESCC (20–25), the evidence of immunotherapy is limited in the neoadjuvant setting. The CSCO guideline mentioned that preoperative neoadjuvant immunotherapy of EC lacks strong evidence and suggested combining with chemotherapy or chemoradiotherapy clinically with 2–4 cycles (9). Several small-sample studies reported preliminary results of 2 cycles of neoadjuvant combinational immunotherapy (26–29). This study was designed to validate immunotherapy in a neoadjuvant setting in the real world with a relatively large sample and further analyze factors that might be associated with efficacy. Besides, 3–4 cycles of neoadjuvant treatment are also common in clinical practice, and these patients were included in this study.

## Methods

### Study Design and Patients

Patients with resectable locally advanced ESCC who received neoadjuvant sintilimab in combination with chemotherapy followed by surgery between July 2019 to August 2021 were identified from the database of Thoracic Surgery, The Fourth Hospital of Hebei Medical University. The inclusion criteria were (1) adult patients (age≥18); (2) histologically confirmed, resectable ESCC with clinical stage II–IVA, determined according to American Joint Committee on Cancer (AJCC) 8th edition TNM staging system; (3) completed neoadjuvant sintilimab with chemotherapy (platinum and taxanes); (4) patients underwent resection; and (5) completeness of full medical records. Patients diagnosed with T4b were excluded.

### Staging

Contrast-enhanced thoracoabdominal CT scan, esophageal enhanced magnetic resonance imaging (MRI), endoscopic ultrasound (EUS), and cervical ultrasound were performed for clinical staging at baseline, every two cycles and before resection. In addition, position emission tomography (PET) was used when necessary. Histopathological tests were carried out for postoperative pathologic staging according to the standard protocols. Clinical and pathological staging was determined according to the AJCC 8th edition TNM staging system.

### Treatment and Follow-Up

Prior to the resection, all patients completed 2–4 cycles (3 weeks/cycle) of treatment of sintilimab (200 mg, I.V., D1) in combination with chemotherapy (platinum and taxanes), followed by examinations such as those given pretreatment for efficacy evaluation. The surgical protocol included McKeown and Ivor Lewis esophagectomy with two- or three-field lymphadenectomy. Follow-up was routinely conducted every 3 months during the first 2 years after surgery, and then every 6 months after 2 years.

### Observation Indices

Pathologic complete response (pCR) was defined as no evidence of residual tumor cells of the complete resected tumor specimen and all sampled regional lymph nodes following completion of neoadjuvant therapy and resection. Major pathological response (MPR) was defined as less than 10% of residual tumor cells within the primary tumor bed after neoadjuvant treatment and resection. R0 resection was defined as a microscopically margin-negative resection in which no gross or microscopic tumor remains in the primary tumor bed. The preoperative clinical stage and postoperative pathological stage were compared to the baseline clinical stage. A reduction in either the T descriptor, the N descriptor, or both was defined as tumor downstaging. Disease-free survival (DFS) was defined as the time from the date of surgery to recurrence or death by any cause. Overall survival (OS) was defined as the time from the date of surgery to death by any cause. Safety outcomes were measured by incidence of surgical complications, which are graded according to the Clavien–Dindo classification system, and the proportion or incidence of treatment-related adverse events (TRAEs), which are graded according to the National Cancer Institute–Common Toxicity Criteria for Adverse Events (NCI-CTCAE, version 5.0).

### Statistical Analysis

Statistical analysis was performed after the completion of data collection and verification. Full patient demographic information and baseline characteristics were tabulated and analyzed. The categorical variables were shown in person count and percentage. Effectiveness and safety were analyzed in all patients and were presented in person count and percentage. Comparisons between the subgroups were performed using chi-square tests or Fisher’s exact test. The median follow-up time was calculated by using the reverse Kaplan–Meier method. DFS and OS were analyzed with the Kaplan–Meier method All statistical testing is two-tailed and performed at the 5% significance level.

## Results

### Baseline Characteristics

Between July 2019 and August 2021, a total of 96 patients with resectable locally advanced ESCC, who met the selection criteria at our center, were reviewed in this study. The major characteristics of the patients are shown in **Table 1**. The cohort was primarily male (*n* = 67, 69.8%) at a median age of 65 years (IQR, 60–69 years). Most patients were diagnosed at stage II–III (*n* = 92, 95.9%) and had an ECOG PS score of 0–1 (*n* = 84, 87.5%). Regarding the preoperative clinical stage, cT3 (*n* = 86, 89.6%) was predominant in T category, while N1 (*n* = 54, 56.3%) was most common in N category followed by N0 (*n* = 37, 38.5%). Tumors were most often found in the middle (*n* = 46, 47.9%) and lower (*n* = 36, 37.5%) parts of the esophagus.

**Table 1:** Patient baseline characteristics.

Characteristics	No. (%)
**Age (years)**	
** Median (IQR)**	65 (60–69)
** <65**	46 (47.9%)
** 65–80**	50 (52.1%)
**Sex**	
** Male**	67 (69.8%)
** Female**	29 (30.2%)
**Smoking**	
** Yes**	36 (37.5%)
** No**	60 (62.5%)
**Alcohol Drinking**	
** Yes**	41 (42.7%)
** No**	55 (57.3%)
**Tumor Location**	
** Upper Esophagus**	14 (14.6%)
** Middle Esophagus**	46 (47.9%)
** Lower Esophagus**	36 (37.5%)
**Clinical TNM Stage**	
** II**	40 (41.7%)
** III**	52 (54.2%)
** IVA**	4 (4.2%)
**Clinical T Stage**	
** 2**	5 (5.2%)
** 3**	86 (89.6%)
** 4a**	5 (5.2%)
**Clinical N Stage**	
** 0**	37 (38.5%)
** 1**	54 (56.3%)
** 2**	5 (5.2%)
**ECOG PS Score**	
** 0**	39 (40.6%)
** 1**	45 (46.9%)
** 2**	12 (12.5%)
Therapeutic regimen*	
** Sintilimab/albumin-bound paclitaxel/nedaplatin**	87 (90.6%)
** Sintilimab/albumin-bound paclitaxel/cisplatin**	4 (4.2%)
** Sintilimab/docetaxel/cisplatin**	5 (5.2%)
**Surgical procedure**	
** Ivor Lewis**	6 (6.3%)
** McKeown**	90 (93.8%)

^*^Sintilimab 200 mg, Albumin-bound Paclitaxel 260 mg/m^2^, Nedaplatin 80 mg/m^2^, Cisplatin 80 mg/m^2^, Docetaxel 75 mg/m^2^, d1, Q3W.

### Neoadjuvant Treatment and Outcome

Forty-eight (50.0%) patients received 2 cycles of the neoadjuvant treatment; another forty-eight (50.0%) patients received 3–4 cycles. As shown in **Table 2**, postoperative pathologic analysis showed that 29 (30.2%) patients achieved pCR (ypT0N0), four patients were ypT0N+ responders, and 60 (62.5%) patients achieved MPR. In addition, 80 (83.3%) patients obtained preoperative clinical downstaging, and 71 (74.0%) achieved postoperative pathological downstaging.

**Table 2:** Response assessment.

	No.(%)
Pathologic Complete Response (pCR)	29 (30.2%, 95% CI, 21.3%–40.4%)
Major Pathologic Response (MPR)	60 (62.5%, 95% CI, 52.0%–72.2%)
Clinical Downstaging	80 (83.3%, 95% CI, 74.4%–90.2%)
Clinical Downstaging in T category	72 (75.0%, 95% CI, 65.1%–83.3%)
Clinical Downstaging in N Category*	42 (71.2%, 95% CI, 57.9%–82.2%)
Pathologic Downstaging	71 (74.0%, 95% CI, 64.0%–82.4%)
Pathologic Downstaging in T category	68 (70.8%, 95% CI, 60.7%–79.7%)
Pathologic Downstaging in N category*	36 (61.0%, 95% CI, 47.4%–73.5%)

^*^At baseline, a total of 59 patients were clinical N+.

### Surgical Treatment

Of the 96 patients, all underwent scheduled surgery, R0 resection was achieved in 95 patients (99%), and one patient had R1 resection because of an intraoperative finding of indistinct limit out of the surrounding tissue. The median interval between the end of neoadjuvant therapy and surgery was 37.5 days (IQR, 32–41 days). The median operation time was 234.5 min (IQR, 214–256 min), and the median intraoperative blood loss was 150 ml (IQR, 100–150 ml). The median length of hospital stay was 12 days (IQR, 10–14 days).

### Subgroup Analysis

The subgroup analysis demonstrated the clinical benefits in favor of the patients who were diagnosed at earlier stages or completed 3–4 cycles of neoadjuvant treatment. The MPR rate of Stage II patients was 77.5% (95% CI, 61.5%–89.2%) compared to 51.8% (95% CI, 38.0%–65.3%) in those with Stage III–IVA diseases (*p* = 0.0114). Consistently, the pCR rate was significantly improved in Stage II patients (45.0%, 95% CI, 29.3%–61.5% vs. 19.6%, 95% CI, 10.2%–32.4%, *p* = 0.0125). Although there was no significant difference in pCR or MPR across clinical T stage groups, better outcomes were noted with the cN0 patients (pCR: 43.2%, 95% CI, 27.1%–60.5% vs. 22.0%, 95% CI, 12.3%–34.7%, *p* = 0.0395; MPR: 78.4%, 95% CI, 61.8%–90.2% vs. 52.5%, 95% CI, 39.1%–65.7%, *p* = 0.0166). Notably, compared to those who received 2 cycles of neoadjuvant treatment, patients who completed 3–4 cycles showed a consistently higher pCR rate (47.9%, 95% CI, 33.3%–62.8% vs. 12.5%, 95% CI, 4.7%–25.2%, *p* = 0.0003), a higher MPR rate (83.3%, 95% CI, 69.8%–92.5% vs. 41.7%, 95% CI, 27.6%–56.8%, *p* < 0.0001), and a higher postoperative pathologic downstaging rate (87.5%, 95% CI, 74.8%–95.3% vs. 60.4%, 95% CI, 45.3%–74.2%, *p* = 0.0047) (see **Table 3**).

**Table 3:** Subgroup Analysis.

		pCR	*P*-value	MPR	*P*-value	Preoperative Clinical Downstaging	*P*-value	Postoperative Pathologic Downstaging	*P*-value
Age (years)	< 65(n=46)	12 (26.1%, 95% CI, 14.3%-41.1%)	0.5054	28 (60.9%, 95% CI, 45.4%-74.9%)	0.8339	37 (80.4%, 95% CI, 66.1%-90.6%)	0.5860	32 (69.6%, 95% CI, 54.2%-82.3%)	0.3635
	65-80 (n=50)	17 (34.0%, 95% CI, 21.2%-48.8%)		32 (64.0%, 95% CI, 49.2%-77.1%)		43 (86.0%, 95% CI, 73.3%-94.2%)		39 (78.0%, 95% CI, 64.0%-88.5%)	
Sex	Male (n=67)	20 (29.9%, 95% CI, 19.3%-42.3%)	>0.999	43 (64.2%, 95% CI, 51.5%-75.5%)	0.6503	55 (82.1%, 95% CI, 70.8%-90.4%)	0.7696	51 (76.1%, 95% CI, 64.1%-85.7%)	0.4605
	Female (n=29)	9 (31.0%, 95% CI, 15.3%-50.8%)		17 (58.6%, 95% CI, 38.9%-76.5%)		25 (86.2%, 95% CI, 68.3%-96.1%)		20 (69.0%, 95% CI, 49.2%-84.7%)	
Smoking	Yes (n=36)	13 (36.1%, 95% CI, 20.8%-53.8%)	0.3643	25 (69.4%, 95% CI, 51.9%-83.7%)	0.3840	31 (86.1%, 95% CI, 70.5%-95.3%)	0.7783	30 (83.3%, 95% CI, 67.2%-93.6%)	0.1495
	No (n=60)	16 (26.7%, 95% CI, 16.1%-39.7%)		35 (58.3%, 95% CI, 44.9%-70.9%)		49 (81.7%, 95% CI, 69.6%-90.5%)		41 (68.3%, 95% CI, 55.0%-79.7%)	
Alcohol Drinking	Yes (n=41)	13 (31.7%, 95% CI, 18.1%-48.1%)	0.8247	26 (63.4%, 95% CI, 46.9%-77.9%)	>0.999	34 (82.9%, 95% CI, 67.9%-92.8%)	>0.999	30 (73.2%, 95% CI, 57.1%-85.8%)	>0.999
	No (n=55)	16 (29.1%, 95% CI, 17.6%-42.9%)		34 (61.8%, 95% CI, 47.7%-74.6%)		46 (83.6%, 95% CI, 71.2%-92.2%)		41 (74.5%, 95% CI, 61.0%-85.3%)	
Tumor Location	Upper (n=14)	7 (50.0%, 95% CI, 23.0%-77.0%)	0.1874	9 (64.3%, 95% CI, 35.1%-87.2%)	>0.999	12 (85.7%, 95% CI, 57.2%-98.2%)	0.5659	13 (92.9%, 95% CI, 66.1%-99.8%)	0.2100
	Middle (n=46)	11 (23.9%, 95% CI, 12.6%-38.8%)		29 (63.0%, 95% CI, 47.5%-76.8%)		12 (85.7%, 95% CI, 57.2%-98.2%)		32 (69.6%, 95% CI, 54.2%-82.3%)	
	Lower (n=36)	11 (30.6%, 95% CI, 16.3%-48.1%)		22 (61.1%, 95% CI, 43.5%-76.9%)		12 (85.7%, 95% CI, 57.2%-98.2%)		26 (72.2%, 95% CI, 54.8%-85.8%)	
Clinical Stage	II (n=40)	18 (45.0%, 95% CI, 29.3%-61.5%)	**0.0125**	31 (77.5%, 95% CI, 61.5%-89.2%)	**0.0114**	34 (85.0%, 95% CI, 70.2%-94.3%)	0.7867	28 (70.0%, 95% CI, 53.5%-82.4%)	0.4867
	III/IVA (n=56)	11 (19.6%, 95% CI, 10.2%-32.4%)		29 (51.8%, 95% CI, 38.0%-65.3%)		46 (82.1%, 95% CI, 69.6%-91.1%)		43 (76.8%, 95% CI, 63.6%-87.0%)	
Clinical T Stage	T2 (n=5)	2 (40.0%, 95% CI, 5.3%-85.3%)	0.8696	3 (60.0%, 95% CI, 14.7%-94.7%)	0.8755	4 (80.0%, 95% CI, 28.4%-99.5%)	0.8287	3 (60.0%, 95% CI, 14.7%-94.7)	0.3753
	T3 (n=86)	26 (30.2%, 95% CI, 20.8%-41.1%)		53 (61.6%, 95% CI, 50.5%-71.9%)		71 (82.6%, 95% CI, 72.9%-89.9%)		63 (73.3%, 95% CI, 62.6%-82.2%)	
	T4 (n=5)	1 (20.0%, 95% CI, 0.5%-71.6%)		4 (80.0%, 95% CI, 28.4%-99.5%)		5 (100%, 95% CI, 47.8%-100%)		5 (100%, 95% CI, 47.8%-100%)	
Clinical N Stage	N0 (n=37)	16 (43.2%, 95% CI, 27.1%-60.5%)	**0.0395**	29 (78.4%, 95% CI, 61.8%-90.2%)	**0.0166**	31 (83.8%, 95% CI, 68.0%-93.8%)	0.3264	26 (70.3%, 95% CI, 53.0%-84.1%)	0.6336
	≥N1 (n=59)	13 (22.0%, 95% CI, 12.3%-34.7%)		31 (52.5%, 95% CI, 39.1%-65.7%)		54 (91.5%, 95% CI, 81.3%-97.2%)		45 (76.3%, 95% CI, 63.4%-86.4%)	
ECOG PS	0 (n=39)	13 (33.3%, 95% CI, 19.1%-50.2%)	0.6533	23 (59.0%, 95% CI, 42.1%-74.4%)	0.6684	33 (84.6%, 95% CI, 69.5%-94.1%)	>0.999	28 (71.8%, 95% CI, 55.1%-85.0%)	0.8151
	≥1 (n=57)	16 (28.1%, 95% CI, 17.0%-41.5%)		37 (64.9%, 95% CI, 51.1%-77.1%)		47 (82.5%, 95% CI, 70.1%-91.3%)		43 (75.4%, 95% CI, 62.2%-85.9%)	
Cycle Numbers	2 (n=48)	6 (12.5%, 95% CI, 4.7%-25.2%)	**0.0003**	20 (41.7%, 95% CI, 27.6%-56.8%)	**<0.0001**	36 (75.0%, 95% CI, 60.4%-86.4%)	0.0528	29 (60.4%, 95% CI, 45.3%-74.2%)	**0.0047**
	3-4 (n=48)	23 (47.9%, 95% CI, 33.3%-62.8%)		40 (83.3%, 95% CI, 69.8%-92.5%)		44 (91.7%, 95% CI, 80.0%-97.7%)		42 (87.5%, 95% CI, 74.8%-95.3%)	

Number less than 0.05 were bolded.

### Follow-Up

**Figure 1:**
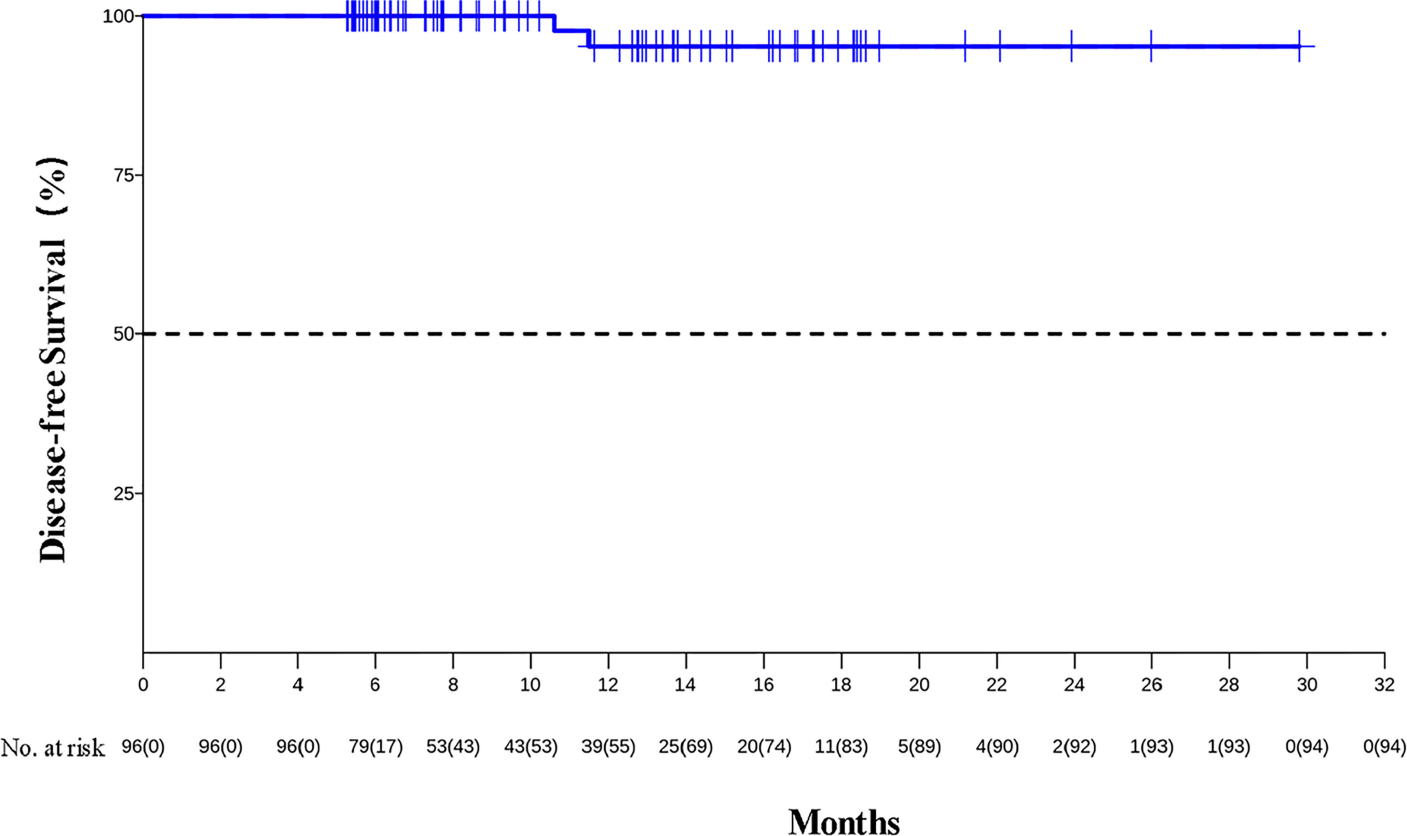
Kaplan–Meier estimates of DFS.

As of data cutoff on December 31, 2021, the median follow-up was 8.9 months (IQR, 6.2 to 14.3 months). The median DFS was not reached, and the 1-year DFS rate was 95.2% (95% CI, 88.8%–100%) (see **Figure 1**). Two patients developed recurrence on 10.6 and 11.5 months after surgery because of live metastasis and lymph node metastasis, respectively. Their clinical stages at baseline were IVA and III. They completed two cycles of neoadjuvant therapy and achieved R0 resection, but did not achieve pCR. There were no deaths reported.

### Safety Profile

Surgical complications are summarized in **Table 4**. No patients experienced intraoperative complications. Grade 1–2 postoperative complications were observed in 45 (46.9%) patients, with the most frequent events being pulmonary infection (26.0%) and arrhythmia (22.9%). Grade 3–4 postoperative complications occurred in two (2.1%) patients. Forty-nine (51.0%) and 12 (12.5%) patients developed grade 1–2 treatment-related adverse events (TRAEs) and grade 3–4 TRAEs, respectively. The most common grade 3–4 TRAEs were neutropenia (8.3%) and leukopenia (3.1%) (**Table 5**).

**Table 4:** Surgical complications.

	Grade 1–2	Grade 3–4
Any Complications	45 (46.9%)	2 (2.1%)
Anastomotic Leakage	5 (5.2%)	0
Arrhythmia	22 (22.9%)	0
Acute Respiratory Failure	3 (3.1%)	1 (1.0%)
Pulmonary Infection	25 (26.0%)	1 (1.0%)

**Table 5:** Treatment-related adverse events.

	Grade 1–2	Grade 3–4
**Any treatment-related adverse event**	49 (51.0%)	12 (12.5%)
Leukopenia	25 (26.0%)	3 (3.1%)
Neutropenia	10 (10.4%)	8 (8.3%)
Anemia	10 (10.4%)	2 (2.1%)
Thrombocytopenia	13 (13.5%)	0
Liver Abnormalities	7 (7.3%)	2 (2.1%)
Cardiotoxicity	1 (1.0%)	0
Pneumonitis	2 (2.1%)	0
Hypothyroidism	8 (8.3%)	2 (2.1%)
Immune-mediated colitis	3 (3.1%)	1 (1.0%)
Sensory neuropathy	17 (17.7)	0
Nausea	13 (13.5%)	0
Diarrhea	6 (6.3%)	0
Alopecia	19 (19.8%)	0
Fatigue	4 (4.2%)	0

## Discussion

Currently, the standard neoadjuvant treatment of locally advanced EC remains platinum and taxane-based chemotherapy or chemoradiation. Immunotherapy, immune checkpoint inhibitors (ICIs) in particular, has provided an immense breakthrough in cancer therapeutics and has become a new pillar to cancer treatment. Several studies have shown that PD-1 inhibitors plus chemotherapy prolonged survival for patients with advanced ESCC (20–25). We thus conducted this study to assess the feasibility and safety of sintilimab plus chemotherapy in patients with resectable local advanced ESCC.

In the present study, neoadjuvant sintilimab plus chemotherapy in patients with resectable locally advanced ESCC produced promising results: Twenty-nine (30.2%) patients achieved pCR, the MPR rate was 62.5%, the clinical downstaging rate was 83.3%, pathological downstaging was achieved in 74.0% of patients, and 1-year DFS rate was 95.2%. Besides, this regimen did show a favorable safety profile in this population. Furthermore, this study demonstrated that patients diagnosed at earlier stages or completed 3–4 cycles of neoadjuvant treatment had a better pCR rate.

In neoadjuvant chemo-immunotherapy settings, several small-sample studies revealed that the pCR rate was 16.7% to 50%, which correlated with different baseline characteristics and drug combinations (26–35). The rate of pCR was much better than that of nCT, reported to be less than 10%, and was comparable to nCRT, reported to be 27.6%–43.2% (15, 36–38). The clinical downstaging rate was 83.3%, significantly higher than that of neoadjuvant chemotherapy (30%) (39). The pathologic downstaging rate was 74.0%, better than that of neoadjuvant chemotherapy (26.1%–63.3%) (40–48). For lung cancer, several large-sample phase 3 trials have extended the neoadjuvant treatment cycle to 3–4 cycles, such as KEYNOTE-671, CheckMate 816, IMpower030, and AEGEAN. Our study shows that patients who completed 3–4 cycles of neoadjuvant treatment tend to have a higher pCR rate, which is worth mentioning in perspective large-sample phase 3 trials of neoadjuvant chemotherapy for ESCC.

However, a propensity score−matched study from the National Cancer Center in China shows that there was no difference in survival between the nCT and nCRT groups (5-year OS rate 77.3% vs. 61.3%, *p* = 0.141), although nCRT correlated to the significantly higher pCR rates (38.9% vs. 5.6%, *p* < 0.001) (38). High postoperative pCR rate of combined radiotherapy did not seem to improve long-term survival. Possible explanations for this phenomenon may be the number of non-cancer-related deaths in the short term, and the frequency of adjuvant therapy use may hinder the detection of potential survival advantage (38, 49, 50). Moreover, some investigators thought it is possible that radiotherapy can increase the local pathological response, but may be poor at controlling occult systemic metastasis (38, 51). Considering this aspect, immunotherapy, as a systemic treatment confirmed to improve long-term survival in patients with advanced ESCC, may be better to translate the pathologic response into a long-term survival benefit. Initial results of a randomized clinical trial to compare the safety and long-term survival of nCRT with that of nCT for patients with locally advanced ESCC showed that the nCRT group had a higher pCR rate (35.7% vs. 3.8%, *p* < 0.001) than the nCT group; 1-year overall survival was 87.1% in the nCRT group and 82.6% in the nCT group (*p* = 0.30) (36). In our study, the median follow-up was 8.9 months, 1-year DFS rate was 95.2%, and no deaths were reported. Three- or 5-year survival rate in the follow-up of these studies will offer conclusive results, that is, whether the better postoperative pathologic response of neoadjuvant chemo-immunotherapy would result in a long-term survival benefit.

The CheckMate 577 clinical trial showed that in patients with resected esophageal or GEJ cancer who had received neoadjuvant chemoradiotherapy and had residual pathological disease, nivolumab adjuvant therapy significantly prolonged disease-free survival compared to placebo (52). Whether patients who achieve pCR after neoadjuvant immunotherapy need adjuvant treatment or simply require regular observation still needs to be determined. Whether PD-1 inhibitor alone or in combination with chemotherapy is the better adjuvant treatment regimen is also not known. In our study, all patients who could tolerate neoadjuvant treatment received adjuvant therapy of sintilimab alone or in combination with chemotherapy, and long-term survival is under follow-up.

No new AEs occurred in this study. The most common grade 3–4 TRAEs were neutropenia and leukopenia, which were mainly caused by chemotherapeutic agents. Furthermore, neoadjuvant chemo-immunotherapy did not increase the degree of difficulty and risk associated with surgery; all patients completed the surgery as planned. The postoperative complications were relatively manageable, and there was no perioperative death. These results validated the manageable safety and feasibility of neoadjuvant sintilimab in combination with chemotherapy in patients with resectable locally advanced ESCC.

Our study may have several inherent limitations. First, it is a retrospective study conducted at a single institution. This may cause biases and affect the power and significance of the finding. Adding a control group of neoadjuvant chemotherapy would decrease bias and be more convincing. However, several clinical trials have investigated chemotherapy alone in the preoperative setting of locally advanced esophageal cancer; the pCR rate was less than 10%, similar to our clinical experience. Second, our study only evaluated short-term efficacy; long-term follow-up (OS and DFS) is necessary to evaluate the long-term clinical benefits of neoadjuvant chemo-immunotherapy for locally advanced ESCC. In the future, prospective cohort studies are worthy of being conducted to gain a better insight into neoadjuvant chemo-immunotherapy in patients with resectable locally advanced ESCC, and to strengthen our findings.

Taken together, our study provided the essential clinical insights into real-world neoadjuvant chemo-immunotherapy for resectable locally advanced ESCC. We have shown that sintilimab combined with chemotherapy was safe and can greatly benefit the clinical outcomes. This combination regimen warrants further exploration in prospective clinical trials.

## Data Availability Statement

The original contributions presented in the study are included in the article/supplementary material. Further inquiries can be directed to the corresponding author.

## Ethics Statement

The studies involving human participants were reviewed and approved by the Ethics Committee of The Fourth Hospital of Hebei Medical. Written informed consent for participation was not required for this study in accordance with the national legislation and the institutional requirements.

## Author Contributions

ZT and HL designed the study. YT and JL collected the data. HL and YT analyzed and interpreted the data. YT, JL, CH, BS, CG, and ZL carried out the clinical treatment and management of the patients. ZT and HL prepared the final draft. All authors contributed to the article and approved the submitted version.

## Funding

This work was funded by the Chinese National Cancer Center (NCC2017A24).

## Conflict of Interest

The authors declare that the research was conducted in the absence of any commercial or financial relationships that could be construed as a potential conflict of interest.

## Publisher’s Note

All claims expressed in this article are solely those of the authors and do not necessarily represent those of their affiliated organizations, or those of the publisher, the editors and the reviewers. Any product that may be evaluated in this article, or claim that may be made by its manufacturer, is not guaranteed or endorsed by the publisher.
